# Comparative Proteomic and Bioinformatic Analysis of the Effects of a High-Grain Diet on the Hepatic Metabolism in Lactating Dairy Goats

**DOI:** 10.1371/journal.pone.0080698

**Published:** 2013-11-19

**Authors:** Xueyuan Jiang, Tao Zeng, Shukun Zhang, Yuanshu Zhang

**Affiliations:** 1 Key Laboratory of Animal Physiology and Biochemistry, Ministry of Agriculture, Nanjing Agricultural University, Nanjing, People’s Repulic of China; 2 College of Animal Science and Technology, Nanjing Agricultural University, Nanjing, People’s Repulic of China; University of South Florida College of Medicine, United States of America

## Abstract

To gain insight on the impart of high-grain diets on liver metabolism in ruminants, we employed a comparative proteomic approach to investigate the proteome-wide effects of diet in lactating dairy goats by conducting a proteomic analysis of the liver extracts of 10 lactating goats fed either a control diet or a high-grain diet. More than 500 protein spots were detected per condition by two-dimensional electrophoresis (2-DE). In total, 52 differentially expressed spots (≥2.0-fold changed) were excised and analyzed using MALDI TOF/TOF. Fifty-one protein spots were successfully identified. Of these, 29 proteins were upregulated, while 22 were downregulated in the high-grain fed vs. control animals. Differential expressions of proteins including alpha enolase, elongation factor 2, calreticulin, cytochrome b5, apolipoprotein A-I, catalase, was verified by mRNA analysis and/or Western blotting. Database searches combined with Gene Ontology (GO) analysis and KEGG pathway analysis revealed that the high-grain diet resulted in altered expression of proteins related to amino acids metabolism. These results suggest new candidate proteins that may contribute to a better understanding of the signaling pathways and mechanisms that mediate liver adaptation to high-grain diet.

## Introduction

Current feeding practices in the dairy industry involve the use of high grain diets, either to meet the energy demands for high milk production, or simply due to shortages in forage-based feed. However, the fermentable carbohydrate in high grain diets increase the rate of fermentation acid production in the rumen, and may lower the rumen pH to acidotic values [1]. Nutrient required for milk synthesis (amino acids, fatty acids, glucose, etc.) must be transported form the rumen and gut to the liver to undergo metabolic conversion. The liver plays a crucial physiological role in the body, and is responsible for many essential metabolic processes. The best-known functions of the liver include bile production, lipid metabolism, hematopoiesis, and glucose metabolism. Liver cells contain many enzymes required for key metabolic interconversions; these enzymes are found within the cytosol, microsomes, mitochondria, or other cellular organelles. The effects of a high-grain diet on liver function ruminants such as cows and goats are not well understood. We hypothesized that a high-grain diet could significantly alter hepatic metabolism. Given the complexity of biological systems, high-throughput profiling is necessary to better understand such global effects. In particular, advances in bioinformatics software and analysis methods have greatly facilitated high-throughput profiling efforts.

Proteomics is an experimental approach that allows for the simultaneous characterization of cellular abundance of hundreds of proteins. Recent progress has made it possible to identify significant global protein expression changes in response to different biological conditions [2], [3], [4], [5]. To date, many studies have focused on characterizing diseased vs. normal liver proteomes [6], [7]. The aim of this study was 1) to determine global protein expression changes in the liver in response to a high-grain diet; and 2) to identify specific targets underlining the well-characterized changes in liver metabolism. Our data may provide valuable hints for uncovering the mechanism of metabolism and signaling of liver in dairy goats, and provide useful information for future studies with cattle.

## Materials and Methods

### 1 Ethics Statement

The animal care and use protocol employed was approved by the Institutional Animal Care and Use Committee of Nanjing Agricultural University and implemented in accordance with the Regulations for the Administration of Affairs Concerning Experimental Animals (China, 1988) and the Standards for the Administration of Experimental Practices (Jiangsu, China, 2008).

### 2 Experimental animals

Ten Saanen dairy goats that had been lactating for 30 d with an average milk production of 1.5 kg/d were used in this experiment. The goats were randomly divided into two groups. One group was fed a control diet comprising 60% forage and 40% mixed concentrate, while the other group received a high-grain diet containing 23% corn, 20% wheat bran, 17% mixed concentrate and 40% forage. Both groups of goats were fed daily at 8:00 and 18:00, and feed was always provided in an amount that met or exceeded the animal’s nutritional requirements. All the goats were housed in individual stalls and had free access to drinking water. After feeding two months on these diets, the goats were sacrificed; liver samples were collected and then snap-frozen in liquid nitrogen.

### 3 Protein sample preparation

All chemicals used for protein separation and extraction were of analytical grade, and MilliQ water was used to prepare all buffers and solutions. Liver tissues were homogenized in an ice-cold buffer (7 M urea, 2 M thiolurea, 2% (w/v) CHAPS, 50 mM dithiothreitol (DTT), 0.8% (v/v) IPG buffer pH 3–10, 1 mM PMSF). The homogenates were swirled for 30 min, followed by a 30 min centrifugation at 15 000 × g at 4°C. The supernatant containing the total liver protein content was pooled and stored at – 80°C until use. The protein concentration was determined using RC DC™(Bio-Rad, USA) kit.

### 4 Two-dimensional gel electrophoresis (2-DE)

2-DE was performed using a 17 cm (nonlinear, pH 3.0–10.0) IPG gel strip (Bio-Rad, USA), according to Chen et al [8], [9], [10]. Total liver protein (850μg) was loaded onto IPG strips using passive rehydration (13 h with 50 V). Isoelectric focusing (IEF) was performed with a voltage gradient of 250 V for 1 h, 500 V for 1 h, 2000 V for 1 h, 8000 V for 3 h, followed by holding at 8000 V until a total of at least 60 000 V-h was reached. Then, the strips were equilibrated for 15 min in a 1% (w/v) DTT-containing equilibration buffer (50 mM Tris-HCl pH 8.8, 6 M urea, 30% (v/v) glycerol and 2% (w/v) SDS) followed by 15 min in 1% (w/v) iodoacetamide in the equilibration buffer. Equilibrated strips were then sealed on the top of 12.5% SDS-PAGE gel for electrophoresis [11]. Gels were stained with 0.08% coomassie brilliant blue (CBB) G-250, and scanned with a high precision scanner (Versa Doc 3000, Bio-Rad). Spot detection, gel matching and interclass analysis were performed using the PDQuest 8.0 software (Bio-Rad). Triplicate replications were carried out for each example. Protein spots were considered to be differentially expressed only if they showed ≥2.0-fold change in intensity, with *P*-value <0.05.

### 5 In-gel digestion and protein identification

Selected gel spots were manually excised and washed twice with MilliQ water. In-gel digestion was performed as described by Katayama [12]. The digested proteins were air-dried and analyzed by using a 4800 MALDI-TOF/TOF Proteomics Analyzer (Applied Biosystems, USA). A protein spot digested with trypsin was used to calibrate the mass spectrometer, using the internal calibration mode [13]. A mass range of 800–3500 Da was used. A combined search (MS plus MS/MS) was performed using GPS Explorert™software v3.6 (Applied Biosystems, USA) and the MASCOT search engine (Matrix Science Ltd., UK), against the NCBI database. Parameter was set as follows: missed cleavage was 1, fixes modification was cysteine carbamidomethylation, variable modification was methionine oxidation, mass tolerance was 0.15 Da for MS and 0.25 Da for MS/MS data. Proteins with a minimum ion score of 95 (*P*<0.05) was considered to be reliably identified.

### 6 Quantitative Real-Time PCR (qRT-PCR)

Total RNA was extracted from each sample using the TRIzol reagent (Invitrogen, USA) according to the manufacturer’s specifications and then reverse-transcribed into cDNA using an oligo(dT)_15_-Primer and M-MLV reverse transcriptase (SunshineBio, Nanjing, China). Gene-specific primers were designed using the Primer 5.0 software (Table 1). Quantitative RT-PCR (qRT-PCR) analysis was performed with MyiQ2 Real-time PCR system (Bio Rad, USA). PCR was performed using iTaq Universal SYBR Green Supermix (Bio Rad, USA), according to the manufacturer’s protocol. Each sample was analyzed in triplicate. For qRT-PCR, the amplifications were performed with the following protocol: 95°C for 1 min, followed by 40 cycles of 95°C for 15 s, 58°C for 30 s. The β-actin gene was used as an internal control [14]. Each sample was first normalized against its β-actin transcript level, and then normalized to the control group. In order to calculate differences in the expression level of each target gene, the 2^−ΔΔCt^ method for relative quantification was used, according to the manufacturer's manual.

**Table 1:** The primer sequences used for quantitative qRT-PCR of the differentially expressed genes related to diet type.

Gene name	Primer Seqience (5′-3′)Sense/antisense	ProductSize(bp)
Alpha enolase	TCGGAGCAGAGGTTTACCACAA GGTTCACTTTCAGCAGGAGGC	286
Elongation factor 2	GCAGTTTGCCGAGATGTATGTG GGTTTGCCCTCCTTGTCCTTA	224
Calreticulin	ACAACAGCCAGGTGGAGTCAGG GGTTTCTTAGCATCAGGGTCAGG	190
Cytochrome b5	GACTCTGATTCAACCATCCCATTC CTGCCCTCCAACACTCACTCTAA	189
Apolipoprotein A-I	ACCTTGGCTGTGCTCTTCCTG CCTCCTCGTGCCACTTCTTCT	285
Superoxide dismutase (Cu-Zn)	GTCTGCGTGCTGAAGGGTGA CTTCCAGCGTTCCCAGTCTTT	307
Catalase	GCCAGCGACCAGATGAAACA CAGCAACAGTGGAGAAGCGAAC	231
*β*-actin	GGGCAGGTCATCACCATT CCGTGTTGGCGTAAAGGT	160

### 7 Western blot analysis

For Western blotting, proteins were extracted from liver samples with RIPA buffer (50 mM Tris pH 7.5, 150 mM NaCl, 1 mM EDTA, 1% TritonX-100, 10% glycerol, 0.5% Sodium deoxycholate, 0.1% SDS, 1 mM PMSF, 10 mg/L aprotinin, 10 mg/L leuprptin); protein qualification was performed using a BCA assay kit (Beyotime, shanghai China). For each sample, 50 µg of total protein and a prestained protein-weight marker (Bio-Rad) were separated on a 10% SDS-PAGE gel and transferred onto a PVDF membrane (0.4 µm, Millipore) in Tris-glycine buffer with 20% (vol/vol) methanol. The membrane was blocked in 5% nonfat milk powder prepared in Tris-buffered saline containing 0.1% Tween 20 for 2 h at room temperature and then incubated with primary antibody overnight at 4°C [15]. The primary antibodies employed were mouse monoclonal anti-calreticulin (BM0344, ABZOOM) used at a 1∶2,000 dilution, and mouse monoclonal anti-apolipoprotein A-I (apoA-I, BM0394, ABZOOM) used at a 1∶2,000 dilution. After several washes with Tris-buffered saline containing 0.1% Tween 20, membranes were incubated in a 1∶20,000 dilution of an anti-mouse horseradish peroxidase-conjugated secondary antibody (Abcam) for 2 h. The signals were detected by chemiluminescence with Clarity Western ECL Substrate (Bio-Rad). The same membrane was incubated with a β-actin antibody as an internal control.

### 8 Bioinformatics analysis

Gene ontology (GO) is widely used to describe protein function in a standardized format. GO analysis of the identified proteins was performed using the Database for Annotation, Visualization and Integrated Discovery (DAVID) annotation tool [16]. GO annotation analysis groups proteins according to their associated biological process (BP), molecular function (MF) and cellular components (CC) annotations. This provides an overview of the main biological processes in which these proteins participate. In addition, we also performed pathway enrichment analysis using the Kyoto Encyclopedia of Genes and Genomes (KEGG) pathway maps.

## Results

### 1 Global identification of differentially expressed proteins

**Figure 1:**
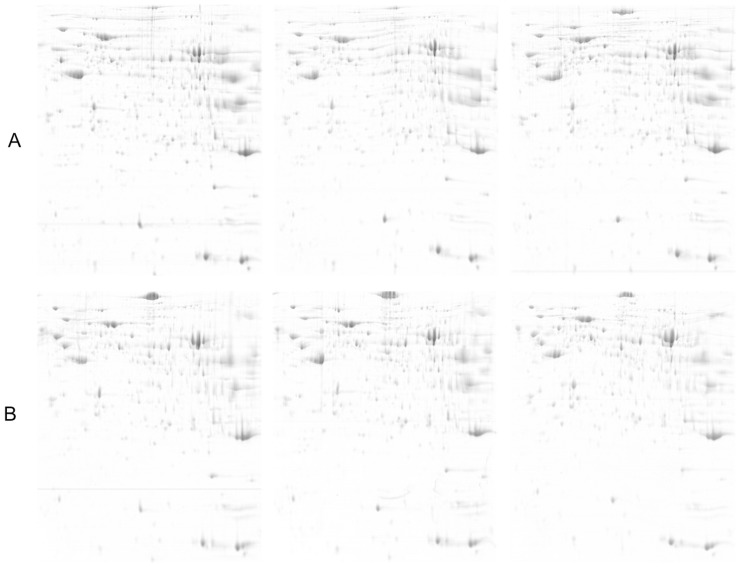
Representative 2-DE images of proteins extracted from dairy goat liver. A) Control group; B) High-grain diet group. Equal amounts of protein (850 µg) were loaded and separated on 17-cm IPG strips (pH 3–10), followed by electrophoresis on 12.5% SDS-PAGE gels for second dimension electrophoresis. The gels were stained with CCB G250. Experiments were performed in triplicate.

**Figure 2:**
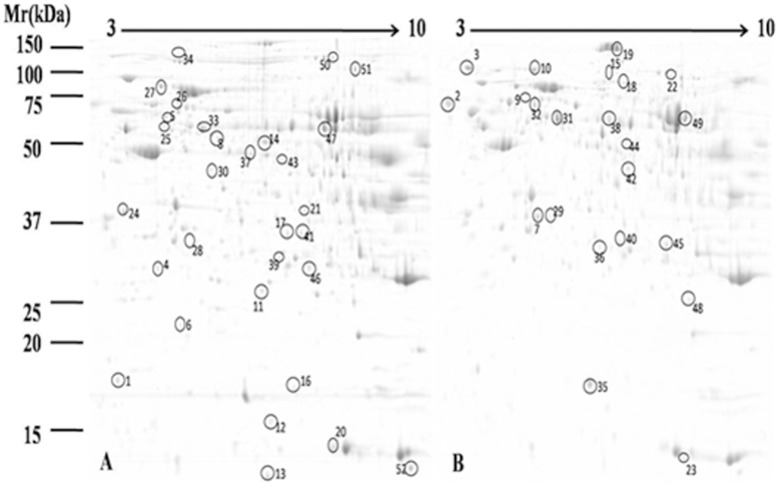
2-DE patterns of proteins extracted from dairy goat liver. A. Control group; B. High-grain diet group. Fifty-two differentially expressed proteins showing significant spot intensity changes are marked in A and B. The proteins to which these 52 differentially expressed protein spots correspond are listed in Table 2.

To understand the influence of diet type on liver metabolism, we employed proteomic tools (2-DE and MALDI-TOF/TOF) to globally identify proteins that were differentially expressed in the liver tissue of goats fed high-grain vs. control diets. To improve resolution and sensitivity, we performed preliminary tests and found that 850 µg protein was the optimal amount for 2-DE analysis. Three replications per sample were sufficient to give highly reproducible results (Fig. 1). The gels were analyzed by PDQuest software (Bio-Rad, USA), which is commonly used in proteomic research. As shown in Fig. 2, 520±18 protein spots were observed on 2-DE gels for control group, as well as 506±31 spots for high-grain diet group.

MALDI-TOF/TOF was used for protein identification. We successfully identified a total of 51 proteins that were differentially expressed in the high-grain group vs. the control group. Of these, 29 proteins showed increased expression and 22 proteins showed decreased expression in the high-grain group (Table 2). Interestingly, a number of proteins including epoxide hydrolase 2 (Figure. 2 and Table 2, spot 9 and spot 32) and superoxide dismutase (Figure. 2 and Table 2, spot 35 and spot 48) were found to be present in more than one spot in the same gel. This is due to differences in pI or molecular weight, and may reflect either a post-translation modification or protein degradation.

**Table 2:** Identification of differentially expressed liver proteins.

aSpot no.	Protein name	Accession No.	Experimental MW(kDa)/pI	bMatched peptides	Score	cFold change
1	cytochrome b5	gi|353817	11.04/5.15	6	495	>2.38
2	calreticulin	gi|545920	46.52/4.31	5	393	>3.37
3	endoplasmin precursor	gi|27807263	92.65/4.76	7	606	>3.37
4	apolipoprotein A-I, apoA-1	gi|245563	28.42/5.57	5	325	>2.10
5	Formimidoyltransferase-cyclodeaminase	gi|329663868	59.55/5.57	5	427	<2.33
6	Retinol-binding protein 4	gi|132403	21.40/5.44	2	202	<3.09
7	3-hydroxyanthranilate 3,4-dioxygenase	gi|115495835	32.70/5.51	4	383	>2.05
8	adenosylhomocysteinase	gi|77735583	48.12/5.88	5	414	>2.03
9	epoxide hydrolase 2	gi|115495833	63.33/5.54	1	107	>2.97
10	aldehyde dehydrogenase family 1 member L1	gi|156718104	99.53/5.53	6	495	>2.09
11	abhydrolase domain-containing protein 14B	gi|157428006	22.56/6.05	7	490	<2.00
12	oxidase IV,cytochrome	gi|223590	10.73/6.46	2	251	<5.74
13	phenylalanine hydroxylase-stimulating protein, pterin-4 alpha-carbinolamine dehydratase, PHS, PCD	gi|298373	11.92/6.31	6	493	<4.48
14	alpha enolase	gi|4927286	47.59/6.44	5	480	>4.45
15	transferrin	gi|209973077	79.78/6.92	4	322	>2.22
16	peroxiredoxin V (PrxV) protein	gi|339522297	23.22/8.29	3	226	<2.09
17	sulfotransferase 1A1	gi|29135333	34.11/6.32	2	162	>2.12
18	radixin	gi|4388775	68.60/5.84	3	200	>2.17
19	carbamoyl-phosphate synthase [ammonia], mitochondrial	gi|300795597	165.83/6.28	6	407	>6.02
20	Hemoglobin subunit beta-A	gi|122540	16.07/6.75	6	515	<4.93
21	glycerol-3-phosphate dehydrogenase [NAD(+)]	gi|78365297	38.24/6.42	7	623	<3.00
22	sarcosine dehydrogenase, mitochondrial	gi|300795914	101.69/7.23	3	285	>2.59
23	beta-globin	gi|164136	15.78/6.43	4	435	>2.83
24	annexin A5	gi|120474983	36.11/4.94	7	640	>2.04
25	cytosolic beta-glucosidase	gi|330864802	53.88/5.37	4	413	<2.05
26	plastin-3	gi|114052248	72.30/5.41	6	469	>2.13
27	heat shock cognate 71 kDa protein	gi|13242237	71.06/5.37	6	677	>2.03
28	17-beta-hydroxysteroid dehydrogenase 14	gi|27807265	28.69/6.19	3	326	<5.29
29	3-hydroxyisobutyrate dehydrogenase, mitochondrial precursor	gi|114052937	35.79/8.38	3	302	<2.01
30	galactokinase	gi|150247075	42.66/5.68	6	390	<3.13
32	epoxide hydrolase 2	gi|115495833	63.33/5.54	2	179	>2.18
33	Aldehyde dehydrogenase 7 family, member A1	gi|86823839	55.86/5.69	4	300	<2.03
34	major vault protein	gi|78369428	99.15/5.45	4	299	<4.76
35	Superoxide dismutase [Cu-Zn]	gi|75061021	15.87/5.85	7	467	<2.11
36	protein ETHE1, mitochondrial precursor	gi|77735641	28.40/6.25	5	371	>2.14
37	4-hydroxyphenylpyruvate dioxygenase	gi|62751490	45.11/6.25	6	557	>2.04
38	selenium-binding protein 1	gi|114051361	53.09/6.03	5	391	>2.15
39	enoyl-CoA hydratase precursor	gi|15982640	28.51/8.72	2	206	<2.03
40	pyridoxine-5'-phosphate oxidase	gi|62460506	30.69/8.16	5	380	>2.01
41	glutathione S-transferase mu 1	gi|122692371	25.82/7.01	2	160	<2.13
42	acetyl-CoA acetyltransferase, cytosolic	gi|115495669	41.69/6.46	7	535	>2.02
43	fumarylacetoacetase	gi|154707900	46.53/6.49	2	217	>2.00
44	elongation factor Tu, mitochondrial precursor	gi|27806367	49.71/6.72	7	699	>2.11
45	hydroxyacylglutathione hydrolase, mitochondrial	gi|78369248	34.41/7.71	2	236	>2.07
46	Triosephosphate isomerase	gi|136062	26.98/6.45	5	385	<2.33
47	glutamate dehydrogenase 1, mitochondrial precursor	gi|4885281	61.70/7.66	6	533	<2.08
48	mitochondrial superoxide dismutase 2	gi|256665379	24.74/8.89	3	199	<2.03
49	catalase	gi|242200439	58.15/6.78	7	662	>2.06
50	elongation factor 2	gi|387049	96.30/6.31	8	633	>2.01
51	aconitate hydratase, mitochondrial precursor	gi|27806769	86.05/8.08	5	514	<2.02
52	chaperonin 10	gi|4008131	10.58/9.44	6	480	<2.17

a Numbering corresponds to the 2-DE gel in Fig.1.

b The total number of identified peptide.

c Increased(>) or decreased(<) compared with the control group.

### 2 Validation of differentially expressed proteins

**Figure 3:**
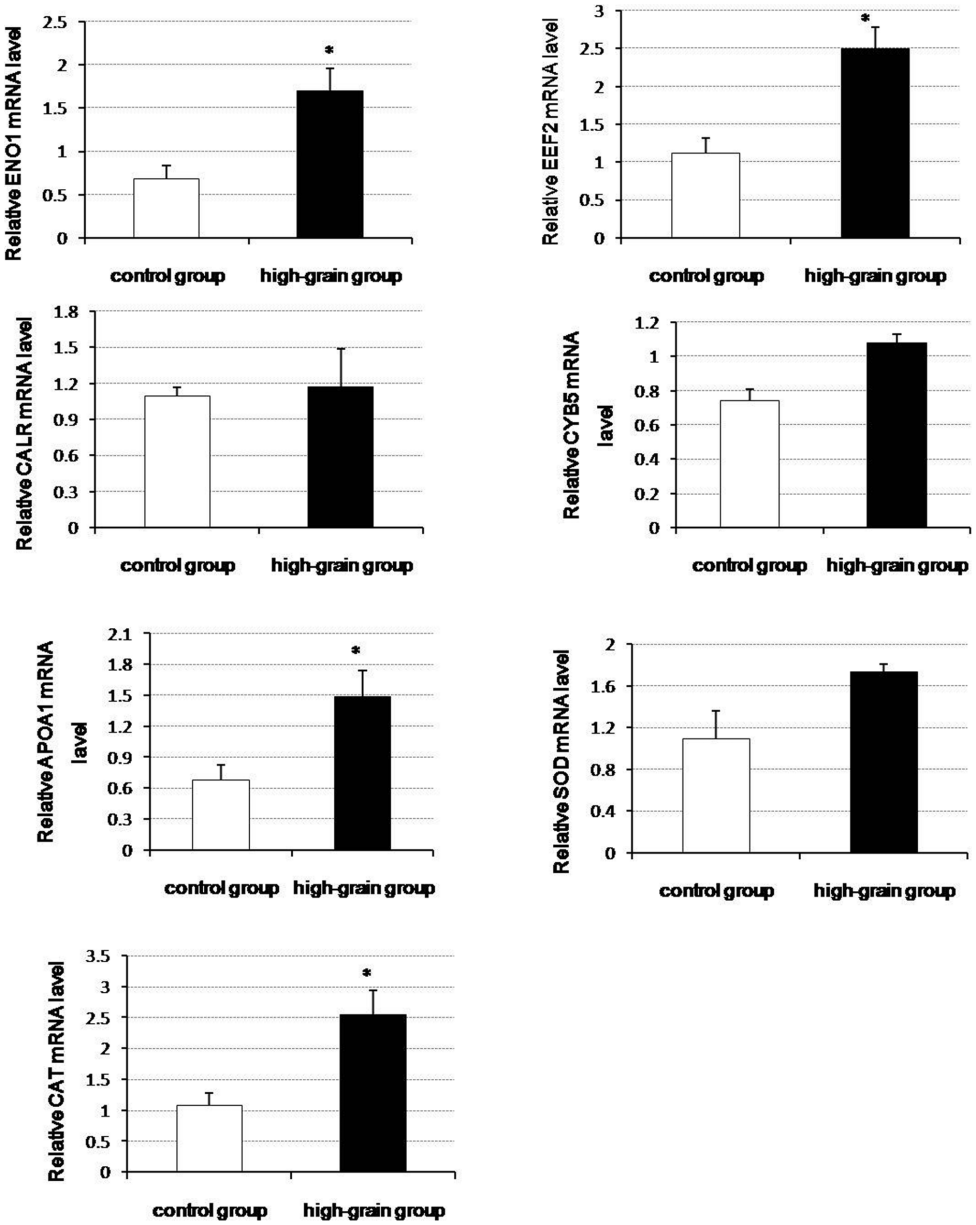
Validation of nine differentially expressed proteins by qRT-PCR analysis. The experimental procedure and the statistical analyses for the parallel runs are described in “Materials and Methods.” Values are presented as means ± SD; n = 5. * indicates *P*<0.05. ENO1: alpha-enolase; EEF2: elongation factor 2; CALR: calreticulin; CYB5: cytochrome b5; APOA1: apolipoprotein A-I; SOD: superoxide dismutase (Cu-Zn); CAT: catalase.

To understand the relationship between changes in the levels of the identified proteins and the transcriptional levels of their encoding genes under different diet type, we performed qRT-PCR analysis for the genes that encode seven of the differentially expressed proteins (Fig. 3). Protein levels do not necessarily correlate well with mRNA expression levels, and such discrepancies are often due to post-transcriptional regulation. It is generally accepted that gene expression does not necessarily predict expression at the protein level [17]. However, changes in the mRNA levels of CYB5 (spot 1), CALR (spot 2), APOA1 (spot 4), ENO1 (spot 14), CAT (spot 49) and EEF2 (spot 50) were found to be consistent with the changes seen in the levels of the corresponding proteins. For proteins such as SOD (spot 35), the observed changes in protein level did not match changes in the mRNA expression level of the corresponding gene.

**Figure 4:**
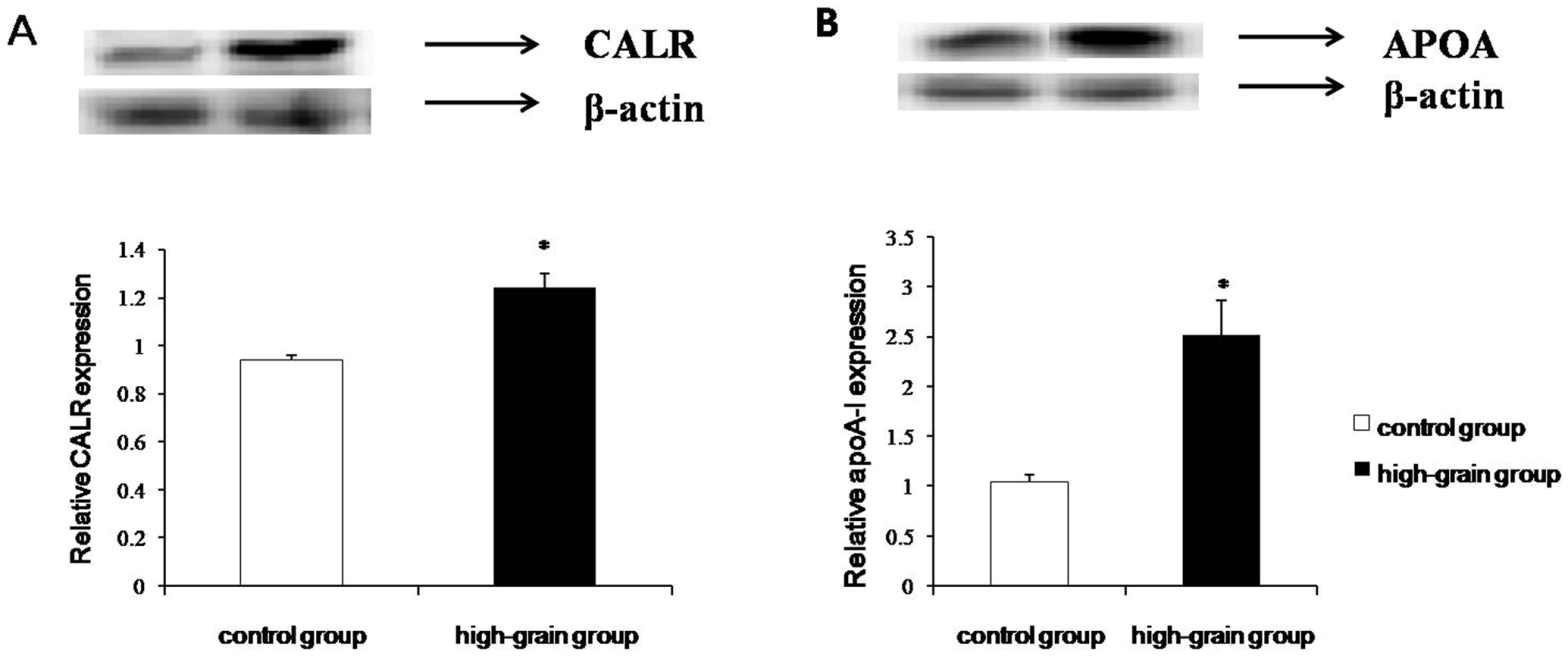
Western blot analysis of calreticulin (CALR; A) and apolipoprotein A-I (ApoA-I; B) in liver tissue samples from high-grain diet and control groups. Protein extracts of liver tissue samples were prepared and subjected to immunodetection with the indicated antibodies. Intensities of CALR and ApoA-I bands were normalized to the corresponding β-actin control. Values are presented as means ± SD; n = 5. * *P*<0.05.

To confirm that the global proteomic changes revealed by 2-DE correspond to change in expression levels of individual proteins, we carried out Western blot analysis of calreticulin and apoA-I. As shown in Fig. 4, there was good agreement between the results for 2-DE and Western blot analysis. Calreticulin detected as a 37-kDa band was significant upregulation in the high grain-fed diet samples compared with controls (Fig. 4A). ApoA-I detected as a single band at 31 kDa, was also significantly upregulated in high grain-fed group samples (Fig. 4B).

### 3 Bioinformatics analysis of differentially expressed proteins

**Figure 5:**
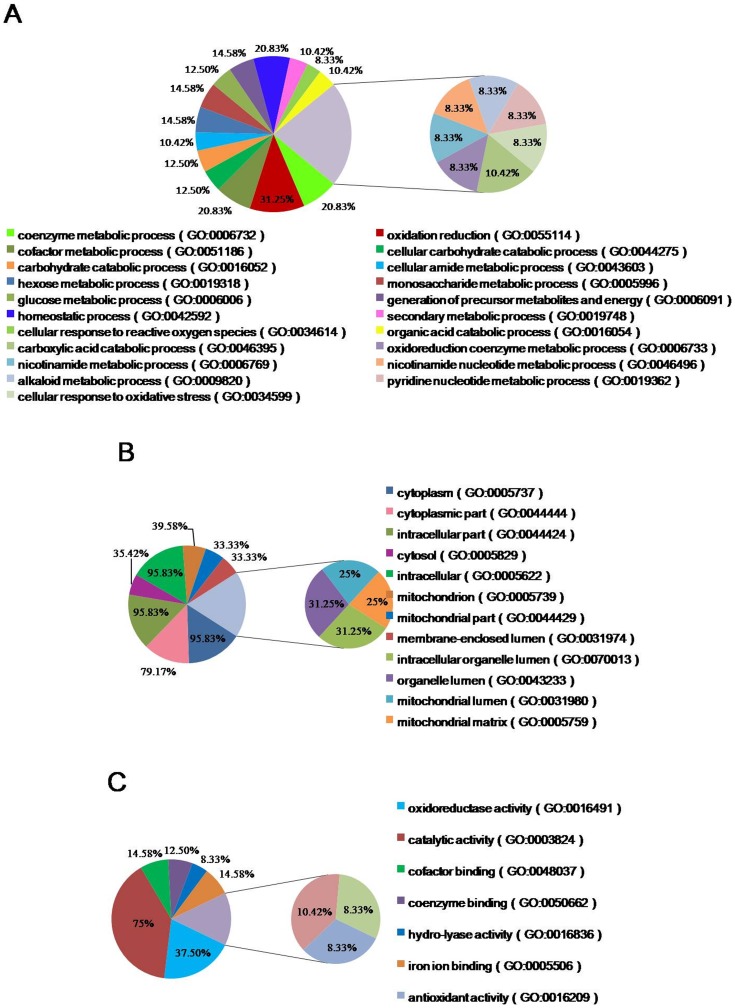
Gene ontology (GO) analysis of differentially expressed proteins. GO annotations are presented by category: A) biological process B) cellular components C) molecular function.

GO analysis is widely used in proteomic research to performed functional annotation of large protein sets, such as those identified by 2-DE. All the proteins that showed ≥2-fold differential expression were included in our GO analysis (Fig. 5). The BP annotation analysis revealed that 31.25% of the identified proteins are involved in oxidation reduction processes, while 20.83% of the proteins were associated with coenzyme metabolic processes, homeostatic processes and cofactor metabolic processes. Another 14.58% of the proteins were involved in hexose metabolic processes, monosaccharide metabolic processes and generation of precursor metabolites and energy. The CC annotation analysis revealed that most of the identified proteins are cytoplasmic and intracellular proteins, while a subset are mitochondrion proteins. The MF annotation analysis revealed that 75% of the identified proteins have catalytic activity, 37.5% of them have oxidoreductase activity, and 26% are coenzyme and cofactors. The remaining proteins have various binding activities. The GO analysis provided an overview of the cellular roles of the differentially proteins, and allowed us to categorize them according to their functions in the context of liver biology.

**Figure 6:**
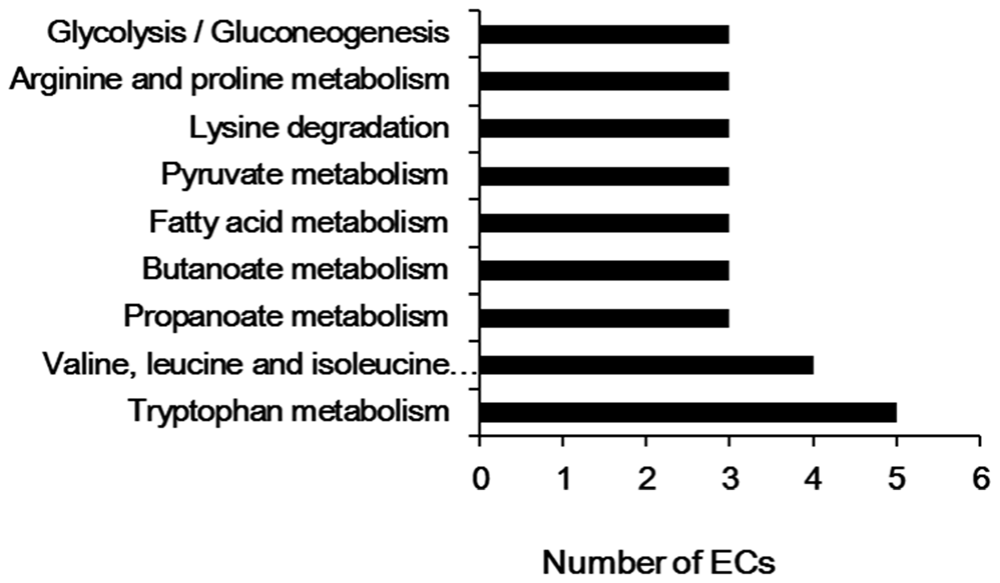
KEGG pathway analysis of differentially expressed proteins. Pathway enrichment analysis was performed using the DAVID web application.

KEGG pathway enrichment analysis is considered one of the most reliable methods for functional annotation. We performed KEGG pathway enrichment analysis on the same set of differentially expressed proteins used for the GO analysis. Among these 51 proteins, 30 proteins were associated with specific KEGG pathways (Fig. 6). As shown in Fig. 6, the differentially expressed proteins were mainly involved in amino acids metabolism, with a subset that was involved in fatty acid metabolism and glycolysis/gluconeogenesis.

## Discussion

In the present study, we carried out proteomic analysis on goat liver samples to globally identify differences in protein expression in animals fed high-grain vs. control diets. Our proteomic data revealed that many interesting proteins were differentially expressed in the two groups. For example, with previous studies demonstrated that many interesting proteins were differentially expressed in the two groups. For example, α-enolase (spot 14) is the glycolytic enzyme that catalyzes the production of phosphoenolpyruvate from 2-phosphoglycerate. Its up-regulation indicated glycolysis in high grain-fed animals. Triosephosphate isomeraser (TPI) (spot 46) participates in glucose catabolism, converting phosphodihydroxyacetone into glyceraldehyde 3-phosphate. Consistent with these changes, GO analysis and KEGG pathway analysis indicated that glucose metabolic processes were significantly altered in the animals fed the high-grain diet.

A number of proteins involved in protein biosynthesis show significant differences in their expression across the two treatments. One of these is Elongation factor Tu (EF-Tu) (spot 44), a GTP-binding protein that is crucial for protein biosynthesis. In the GTP-bound form of the molecule, EF-Tu binds tightly to aminoacyl-tRNA, forming a ternary complex that interacts with the ribosomal acceptor site. During this interaction, GTP is hydrolyzed, and EF-Tu-GDP is released. Another protein involved in protein synthesis is elongation factor-2 (EF-2) (spot 50).This protein catalyzes the translocation of peptidyl-tRNA on the ribosome [18]. EF-2 is involved in translocation step of the eukaryotic polypeptide chain elongation, and selectively binds to the pretranslocational ribosome. Calreticulin (spot 2) is a major endoplasmic reticulum Ca^2+^ binding chaperone with multiple functions. Calreticulin is involved in a variety of cellular signaling pathways, such as those linked to innate immunity, adipocyte differentiation, apoptosis, and cellular stress responses [19], [20]. It also plays a crucial role in regulating intracellular Ca^2+^ homeostasis [21], [22] and steroid-sensitive gene expression [23], [24]. Calreticulin is a lectin-like chaperone and, together with calnexin, plays an important role in quality control during protein synthesis, folding, and pos-ttranslational modification. Calreticulin binds Ca^2+^ and affects cellular Ca^2+^ homeostasis. The protein increases the Ca^2+^ storage capacity of the endoplasmic reticulum and modulates the function of endoplasmic reticulum Ca^2+^-ATPase. Interestingly, it has been reported that enolase 1 (spot 14) can regulate the differentiation and function of mouse mast cells with calreticulin, which was also found in our study [25]. Up-regulation of these proteins is likely to increase the protein synthesis capacity of cell. Consistent with these changes, GO analysis and KEGG pathway analysis also indicated upregulation of protein biosynthesis and amino acids metabolism. The identification of these proteins provides a starting point for further research on metabolic pathways that may be relevant to these conditions.

Other differentially expressed proteins were associated with lipid metabolism. For example, spot 1 was identified as cytochrome *b*5, a heme protein found in the microsomal fraction of liver tissue homogenates. Cytochrome *b*5 is known to function as an electron transfer component in a number of oxidative pathways, including the anabolic metabolism of fats and steroids. It interacts specifically with an NADH-dependent flavoprotein, cytochrome *b*5 reductase, which catalyzes the reduction of the cytochrome by NADH. The cytochrome *b*5 electron-transport system has recently been implicated in fatty acid desaturation reactions in isolated microsomes [26], [27]. Apolipoprotein A-I (apoA-I, spot4) is the principal protein found in high density lipoprotein particles (HDL) that participate in the transport of cholesterol and other lipids. ApoA-I also has a physiological role as an activator of lecithin-cholesterol acy1transferase, an enzyme that plays an important role in lipoproteins metabolism [28], [29]. In our study, the high-grain diet induced the expression of cytochrome *b*5 and apoA-I, suggesting a tendency to increase fatty acid metabolism.

The generation of reactive oxygen species (ROS) is often the first detectable response to abiotic or biotic stress in the body. High levels of ROS can create oxidative stress and damage cellular components. However, lower levels may trigger signaling, allowing the cell to respond to the oxidative stress [30]. Previous studies have identified various antioxidant enzymes involved in ROS metabolism, some of which were found to be differentially expressed in our samples. These include superoxide dismutases (SOD) (spot 35, 48), Glutathione *S*-transferase (GST) (spot 41) and catalase (CAT) (spot 49). Cu/Zn-SOD, a product of the SOD1 gene, is a major Cu-containing enzyme that plays an essential role in alleviating oxidative stress by scavenging superoxide anions. Cu/Zn-SOD suppresses metal-catalyzed hydroxyl radical production [31]. GST is a family of enzymes that plays an important role in protecting cells from cytotoxic oxidation. Our results showed that SOD and GST were both downregulated in high-grain diet group. GO analysis also revealed some differentially expressed liver proteins to have roles in redox process.

Another identified important redox-related protein was glycerol-3-phosphate dehydrogenase (GPDH) (spot 21). The oxidation of glycerol-3-phosphate by mitochondrial GPDH is a major pathway for transfer of cytosolic reducing equivalents to the mitochondrial electron transport chain. Glycerol-3-phosphate is an important intermediate in both lipid and carbohydrate metabolism. Mitochondrial oxidation of glycerol-3-phosphate is linked to the generation of H_2_O_2_ and superoxide (likely via SOD activity) [32]. However, further research will be needed to elucidate the molecular mechanism involved.

GO annotation analysis provided an overview of the functions of the differentially expressed liver proteins. The results showed that the most strongly enriched biological processes were of three main categories: metabolic process, catabolic processes and oxidation reduction. The MF annotation analysis revealed that 75% of the identified proteins were catalytic proteins, 37.5% of them were oxidoreductase, and 26% were coenzyme and cofactors. For the CC category, over 95% of the proteins were cytoplasm proteins, over 30% of them were found in mitochondrion and 33% of them were membrane proteins. Taken together, these results suggest that the identified proteins could play roles in membrane proteins biosynthesis, and/or signal transduction-related pathways. KEGG is a knowledge base for systematic analysis of gene functions, linking genomic information with higher order functional information that is stored in the PATHWAY database [33]. KEGG pathway analysis of the identified proteins revealed that a number of these proteins were related to amino acid metabolism. However, many of the proteins could not be mapped to KEGG pathways; this low mapping rate may be due to lack of available annotations for primary and secondary metabolic pathways in liver. The remaining unmapped proteins could be investigated via analysis of protein-protein interactions, or further database searches. It is likely that further experimental analysis will be needed to elucidate the pathways involved.

Finally, as other studies have noted, changes in protein levels do not necessarily correlate well with changes in mRNA levels; such discrepancies are often attributed to post-transcriptional regulation. It is generally accepted that gene expression does not necessarily predict the expression level of proteins [17]. However, for calreticulin and apoA-I, two of the differentially expressed proteins identified in this study, we observed mRNA expression level changes that were consistent with the changes in levels of the corresponding proteins. A major bottleneck in proteomic studies on goat tissues is the limited availability of suitable antibodies. This limited the number of identified proteins that we could validate by western blotting. Therefore, the combination of three techniques used in this study (2-DE, immunoblotting, and qRT-PCR) allowed us to reliably identify proteins in goat liver that were differentially regulated in response to diet.
